# Altered expression of Tim family molecules and an imbalanced ratio of Tim-3 to Tim-1 expression in patients with type 1 diabetes

**DOI:** 10.3389/fendo.2022.937109

**Published:** 2022-07-28

**Authors:** Yikai Liu, Zhiying Chen, Yang Xiao, Hongzhi Chen, Zhiguang Zhou

**Affiliations:** Key Laboratory of Diabetes Immunology, National Clinical Research Center for Metabolic Diseases, Ministry of Education, and Department of Metabolism and Endocrinology, The Second Xiangya Hospital of Central South University, Changsha, China

**Keywords:** Tim-1, Tim-3, Tim-4, type 1 diabetes, T cell subset-specific transcription factors

## Abstract

**Background:**

T-cell immunoglobulin and mucin domain (Tim) proteins are immunomodulatory molecules that play key roles in the regulation of T-cell activation. Published studies have reported that Tim molecules are involved in the pathogenesis of certain autoimmune diseases. Type 1 diabetes (T1D) is an autoimmune disease in which T cells mediate the destruction of islet β cells. However, the expression of Tim molecules in T1D remains unclear. In this study, we measured the expression of Tim family molecules as well as T-cell subset-specific transcription factors in T1D patients, and we explored the possible involvement of Tim molecules in the pathogenesis of T1D.

**Methods:**

Ninety T1D patients, Thirty-six type 2 diabetes (T2D) patients and forty healthy controls (HCs) were recruited for this study. Peripheral blood mononuclear cells (PBMCs) were isolated, RNA was extracted from the PBMCs and reverse transcribed into cDNA, and gene expression patterns were analysed by RT–qPCR. The expression of Tim molecules in different T-cell subsets was analysed by flow cytometry.

**Results:**

Compared with that in HCs, the mRNA expression of Tim-1 and RORC was increased in T1D patients (*P*=0.0355 and *P*=0.0423, respectively), while the expression of Tim-3 was decreased (*P*=0.0013). In addition, compared with HCs, the ratio of Tim-3 to Tim-1 expression in diabetic patients was decreased (*P<*0.0001 for T1D and *P=*0.0387 for T2D). The ratios of T-Bet to GATA3 expression and RORC to FOXP3 expression were higher in T1D patients than in HCs (*P=*0.0042 and *P=*0.0066, respectively). Furthermore, the T1D patients with defective islet function had more significant imbalances in the Tim-3/Tim-1 and RORC/FOXP3 ratios (*P*<0.0001, and *P*=0.001, respectively). Moreover, Both Tim-3 expression in CD4^+^ T cells and the Tim-3 to Tim-1 ratio were elevated in T1D in the remission phase compared to T1D.

**Conclusion:**

Our study revealed altered expression of Tim molecules in T1D patients. The imbalanced ratios of Tim-3/Tim-1 expression were more pronounced in T1D patients with defective islet function. However, alterations in Tim molecule expression are mitigated in T1D in the remission phase. All these findings suggest that Tim family molecules may be involved in the pathogenesis of T1D.

## Introduction

Type 1 diabetes (T1D), also known as insulin-dependent diabetes, is a common autoimmune disease. In T1D, the insulin-producing cells of the pancreas are destroyed by T lymphocytes, resulting in an absolute lack of insulin in patients (1). Although T1D occurs mostly in children and adolescents, new evidence suggests that the incidence of adult-onset T1D may be underestimated (2, 3). The onset of T1D is relatively rapid, and ketoacidosis can occur in severe cases and can even be life-threatening. At present, the pathogenesis of T1D is not fully understood. It mainly includes genetic and environmental factors, both of which increase the risk of T1D by causing immune imbalance (4, 5). Imbalance of T-cell immune homeostasis is a major factor in the development and progression of autoimmune. Therefore, determining the cause of immune imbalance is significant for the effective treatment of T1D, and this is an urgent problem that needs to be solved.

Members of the T-cell immunoglobulin and mucin domain (Tim) molecule family have been shown to be important regulators of the immune response (6). As the name suggests, Tim molecules were originally identified in T cells, and they mainly regulate the responses of T helper (Th) cells (7). In humans, the Tim family is composed of three members (Tim-1, Tim-3, and Tim-4). The genes that encode the Tim molecules are all located on chromosome 5q33.2 (8). All the Tim molecules contain an IgV domain, a mucin domain, a transmembrane domain, and an intracellular domain, which are considered to play critical roles in maintaining immune homeostasis (9). Tim-1 is preferentially expressed on Th2 cells, where it serves as an effective costimulatory molecule for T-cell activation (10). Tim-3 was first identified on Th1 cells (11). As an inhibitor of inflammatory Th1 cells, Tim-3 interacts with its ligand to promote the death of Th1 cells, thereby reducing the production of interferon-γ (IFN-γ). Tim-4 is mainly expressed on antigen-presenting cells. As a costimulatory molecule for T-cell activation, Tim-4 plays an important role in the activation and development of T cells (12), which can promote the development of Th2 cells and inhibit naive CD4^+^ T cells (13).

Tim family molecules are mainly involved in the regulation of T cells. Activated CD4^+^ T cells may differentiate into several subgroups of cells that perform different functions; these cell types include Th1, Th2, Th17 and regulatory T cells (Tregs) (14). The imbalances in Th-cell subsets and their cytokine production are thought to play essential roles in the pathogenesis of T1D (15).

Herein, we hypothesize that Tims (Tim-1, Tim-3, and Tim-4) may be involved in the pathogenesis of T1D. We evaluated the expression of Tims and T-cell subset-specific transcription factors in T1D patients to elucidate the role of Tim molecules in T1D.

## Materials and methods

### Subjects and clinical parameters

All the patients involved in this study were recruited from the Second Xiangya Hospital. The study included ninety T1D patients, thirty-six T2D patients and forty HCs. T1D was diagnosed based on the World Health Organization (WHO) criteria from 1999 (16): dependence on insulin therapy at disease onset; positive test results for at least one classic islet-specific autoantibody (glutamate acid decarboxylase antibody [GADA], zinc transporter 8 antibody [ZnT8A] or insulinoma-associated protein-2 antibody [IA-2A]), or insufficient C-peptide secretion. The diagnostic criteria for T2D were as follows: insulin resistance and insufficient insulin secretion; negative test results for any pancreatic islet autoantibodies; and no immediate need for insulin treatment. The exclusion criteria for the enrolment of HCs were inflammation and autoimmune diseases. The general demographic information and clinical parameters were obtained from hospital records. The details of all the subjects are summarized in **Table 1**.

**Table 1:** Clinical characteristics of all the study participants.

Variable	T1D (n=90)	T2D (n=36)	HCs (n=40)
Sex (male/female)	59/31	23/13	26/14
Age (years)	25.59 ± 11.96^####^	44.69 ± 7.82	28.25 ± 7.62^####^
BMI (kg/m^2^)	20.71 ± 3.17^####****^	24.09 ± 3.17	23.72 ± 3.04
Duration (months)	27.68 ± 30.27	46.39 ± 48.62	NA
FBG (mmol/L)	7.10 (5.68-10.12)^****^	8.63 (7.11-10.03)^****^	4.71 (4.34-4.97)
FCP (mmol/L)	116.0 (49.08-190.5)^####****^	501.6 (359.7-660.2)	357.1 (258.6-381.6)
PCP (mmol/L)	249.9 (67.8-481.6)^####^	1175 (621.5-1501)	NA
HbA1c (%)	7.87 ± 2.12^****^	8.09 ± 1.91^****^	5.27 ± 0.26
TG (mmol/L)	0.73 (0.59-1.11)^####*^	1.90 (1.21-3.4)^**^	1.00 (0.70-1.83)
TC (mmol/L)	4.55 ± 1.10	4.89 ± 0.89	4.70 ± 1.04
LDL-C (mmol/L)	2.93 ± 0.88	2.90 ± 0.80^*^	3.00 ± 0.93
HDL-C (mmol/L)	1.55 ± 0.40^####***^	1.18 ± 0.35	1.29 ± 0.28
GADA	60/90 (66.7%)	NA	NA
IA-2A	55/90 (61.1%)	NA	NA
ZnT8A	34/90 (37.8%)	NA	NA

The data are expressed as the mean ± standard deviation or as the median of the 25th-75th percentile in parentheses. NA, not applicable. ^##^*P*<0.01, compared with T2D patients. ^####^ *P*<0.0001, compared with T2D patients. *P<0.05, compared with HCs. **P <0.01, compared with HCs. ****P<0.0001, compared with HCs.

This study was approved by the Ethics Committee of Second Xiangya Hospital, Central South University, and informed consent was obtained from all the patients before the study.

### Autoantibodies

GADA, IA-2A, and ZnT8A levels were measured by radiobinding assays (17, 18), which were validated by the Diabetes Antibody Standardization Program (International Association for the Study of Pain [IASP] 2012), and the cut-off values for positivity were 18 units/mL, 2.7 units/mL and 0.011 (ZnT8A index), respectively.

### Isolation of RNA from PBMCs

Blood samples were collected from patients and HCs into heparin sodium tubes, and PBMCs were isolated by Ficoll-Paque density gradient centrifugation. TRIzol reagent (Invitrogen, Thermo Fisher Scientific, Shanghai, China) was used to harvest total RNA from the PBMCs, and the RNA concentration was quantified by measuring the ultraviolet absorbance at 260 nm. Equal amounts of RNA (1μg) were converted to complementary deoxyribonucleic acid (cDNA) with a Thermo Scientific RevertAid First-Strand cDNA Synthesis Kit (Thermo Scientific, Waltham, MA, USA) according to the manufacturer’s instructions.

### Real-time quantitative reverse transcription-polymerase chain reaction (RT–qPCR)

The synthesized cDNAs were amplified with oligonucleotides specific for Tim molecules (Tim-1, Tim-3, and Tim-4), T box expressed in T cells (T-Bet), GATA binding protein 3 (GATA3), forkhead box protein 3 (FOXP3) and related orphan receptor C (RORC) with the SYBR Green kit (Yeasen Biotechnology, Shanghai, China). Quantitative real-time PCR was performed on an ABI PRISM Step One Sequence Detection System (Applied Biosystems, Carlsbad, CA, USA) with the following PCR protocol: in the two-step PCR method, predenaturation was performed by heating at 95°C for 20 seconds, followed by denaturation at 95°C for 1 second and then annealing and extension at 60°C for 20 seconds. The denaturation, annealing, and extension steps were repeated for 40 cycles. Amplification was performed in a total volume of 20 μl containing 2 μg of total cDNA, 0.4 μl of 10 nmol/L primer, 10 μl of SYBM Green mix and 7.2 μl of ddH_2_O. T-cell subset-specific transcription factors expression was measured relative to messenger RNA (mRNA) gene expression; the transcription factors associated with each T-cell subset were as follows: Th1=T-Bet, Th2=GATA3, Th17=RORC, and Treg=FOXP3. All the relative mRNA expression levels of the target genes were calculated as the fold change relative to the GAPDH mRNA expression levels (endogenous control) by using the 2^-ΔΔCT^ method (19). All the primer sequences are listed in **Table 2**.

**Table 2:** Forward and reverse primers for the RT–PCR experiments in this study.

Gene	Forward primer	Reverse primer
GAPDH	TGTTGCCATCAATGACCCCTT	CTCCACGACGTACTCAGCG
Tim-1	GGTCCATCTGTCACACTACCC	CGTGGGTTCCATTGGTCCAG
Tim-3	CCCAGGGAAAAACGAAGTGC	AGCTTCAGTTTGGTCCACGA
Tim-4	CTAACCCCAAGCACCCTTCC	GCTGTATCAGATGCTTTGGATGTC
T-Bet	ATTGCCGTGACTGCCTACCAGA	GGAATTGACAGTTGGGTCCAGG
GATA-3	ACCACAACCACACTCTGGAGGA	TCGGTTTCTGGTCTGGATGCCT
RORC	AAGTGGTGCTGGTTAGGATGTG	GGAGTGGGAGAAGTCAAAGATGG
FOXP3	ATTGAGTGTCCGCTGCTTCCT	ATTGCCGTGACTGCCTACCAGA

### Flow cytometry analysis

To detect the expression of Tims in various T-cell subpopulations, PBMCs were surface stained with the following anti-human mAbs: CD3 Pacific Blue, CD4 FITC, CD8 PerCP-Cy5.5, Tim-1 PE and Tim-3 BB515 (BioLegend, San Diego, CA, USA). Dead cells were excluded from analysis by staining with the Fixable Viability Stain 780 (BD, Franklin lakes, NJ, USA) and analysed using FlowJo Software X (Tree Star, Ashland, OR, USA). The gating strategy used for T-cell subsets is shown in **Figure S1**.

### Statistical analysis

The data are presented as the mean ± standard error unless otherwise stated. Multiple groups were compared by one-way analysis of variance (ANOVA) using the Statistical Package for the Social Sciences (SPSS) software version 18 (IBM Corp, Armonk, NY, USA). Correlations between mRNA expression and clinical parameters were analysed with Spearman’s or Pearson’s rank test, and *P<*0.05 was considered to indicate a significant difference.

## Results

### Demographic and clinical characteristics

The clinical characteristics of the subjects in each group are summarized in **Table 1**. There were no significant differences in sex among the three groups.

### Differences in the mRNA expression levels of Tims and T-cell subset-specific transcription factors among T1D patients, T2D patients and HCs

**Figure 1:**
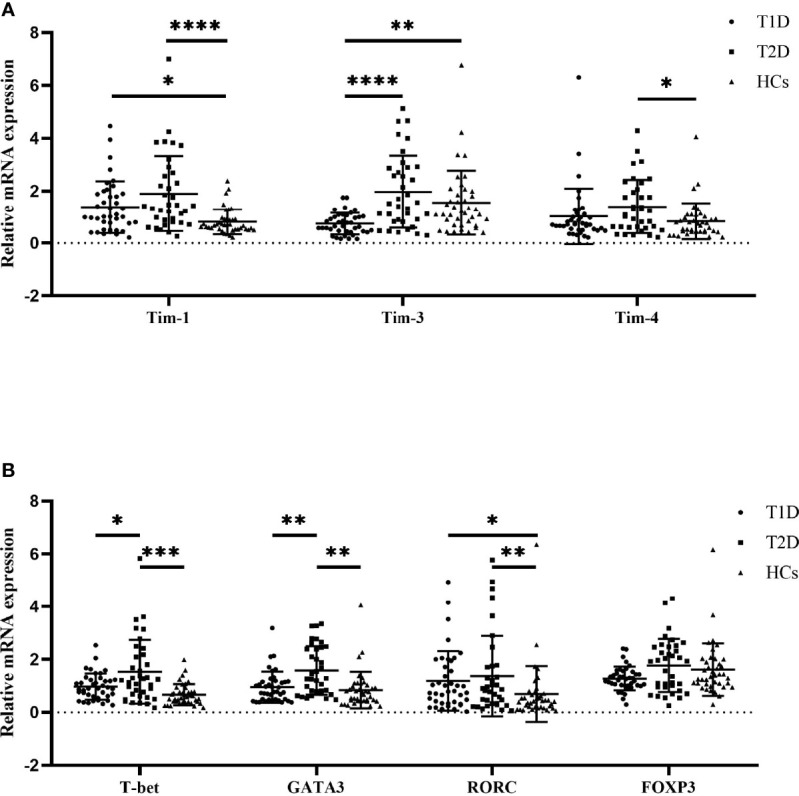
The mRNA expression levels among T1D patients (n=40), T2D patients (n=36) and HCs (n=40) as quantified by RT–qPCR. **(A)** Tims mRNA expression in PBMCs from T1D patients, T2D patients and HCs. **(B)** T-cell subset-specific transcription factor mRNA expression in PBMCs from T1D patients, T2D patients and HCs. The error bars represent the standard deviation of the mean. *P<0.05; **P<0.01; ***P<0.001; ****P<0.0001.

We examined the Tim mRNA expression levels in T1D patients, T2D patients and HCs. As shown in **Figure 1**, compared to that in the HCs, the mRNA expression level of Tim-1 in the T1D patients was increased (0.81 ± 0.47 vs. 1.34 ± 1.01, *P*=0.0355), while the expression level of Tim-3 was decreased (1.55 ± 1.22 vs. 0.74 ± 0.41, *P*=0.0013). The mRNA expression levels of Tim-1 (1.89 ± 1.43 vs. 0.81 ± 0.47, *P*<0.0001) and Tim-4 (1.39 ± 1.02 vs. 0.84 ± 0.69, *P*=0.0454) in the T2D patients were significantly higher than those in the HCs. In addition, the expression level of Tim-3 in the T1D patients was significantly lower than that in the T2D patients (1.55 ± 1.22 vs. 1.96 ± 1.38, *P*<0.0001) (**Figure 1A**). We then explored the mRNA expression levels of T-cell subset-specific transcription factors. The results showed that the mRNA expression of RORC was elevated in the T1D patients compared to the HCs (1.18 ± 1.12 vs. 0.69 ± 1.05, *P*=0.0423), and the mRNA expression of T-bet (0.66 ± 0.40 vs. 1.53 ± 1.21, *P*=0.0001), GATA3 (0.93 ± 0.30 vs. 1.57 ± 0.91, *P*=0.0015) and RORC (0.69 ± 1.05 vs. 1.37 ± 1.52, *P*=0.0040) was increased in the T2D patients. Moreover, T-bet (1.53 ± 1.21 vs. 0.97 ± 0.49, *P*=0.0232) and GATA3 (1.57 ± 0.91 vs. 0.95 ± 0.59, *P*=0.0089) mRNA expression was decreased in the T1D patients compared to the T2D patients (**Figure 1B**).

### The balance of Tim molecules and T-cell subset-specific transcription factor expression among T1D patients, T2D patients and HCs

**Figure 2:**
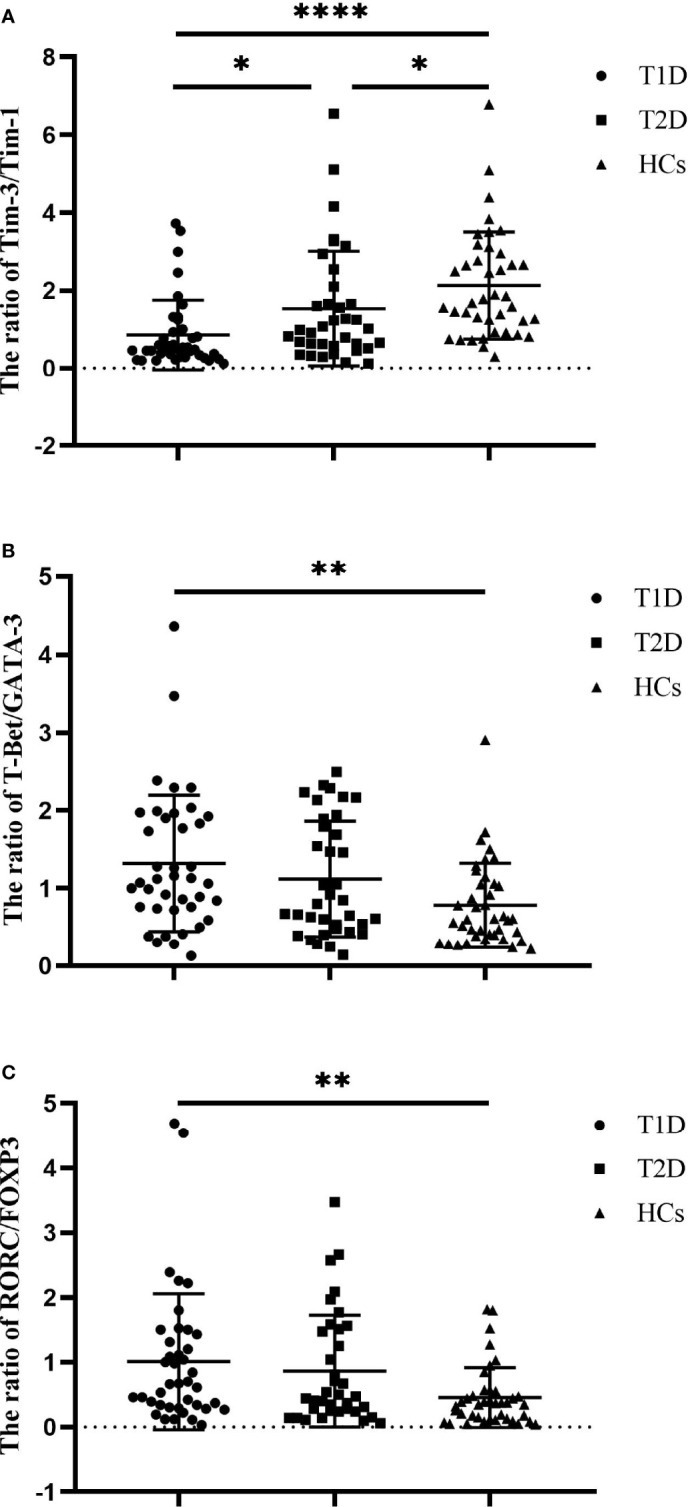
The ratios of Tim molecules and T-cell subset-specific transcription factor expression in T1D patients (n=40), T2D patients (n=36) and HCs (n=40). **(A)** The ratio of Tim-3 to Tim-1 expression in T1D patients, T2D patients and HCs. **(B)** The ratio of T-Bet to GATA3 expression in T1D patients, T2D patients and HCs. **(C)** The ratio of RORC to POXP3 expression in T1D patients, T2D patients and HCs. The error bars represent the standard deviation of the mean. *P<0.05; **P<0.01; ****P<0.0001.

We also compared the balance of the expression of Tim molecules and T-cell subset-specific transcription factors in the diabetic patients and HCs. The results showed that the ratio of Tim-3 to Tim-1 expression in both the T1D (0.85 ± 0.89 vs. 2.13 ± 1.38, *P*<0.0001) and T2D patients (1.54 ± 1.48 vs. 2.13 ± 1.38, *P*=0.0387) was significantly lower than that in the HCs. In addition, the Tim-3 to Tim-1 expression ratio in the T1D patients was decreased compared to that in the T2D patients (0.85 ± 0.89 vs. 1.54 ± 1.48, *P=*0.0225) (**Figure 2A**). The T-Bet to GATA3 expression ratios of the T1D patients were increased compared to those of the HCs (0.78 ± 0.54 vs. 1.32 ± 0.88, *P*=0.0042). However, no statistically significant difference was observed between the T1D and T2D patients (**Figure 2B**). There was an elevated ratio of RORC to FOXP3 expression in the T1D patients compared with the HCs (0.45 ± 0.46 vs. 1.01 ± 1.05, *P=*0.0066). The ratio of RORC to FOXP3 expression in the T2D patients showed an increasing trend, but there was no significant difference between the T2D patients and HCs (*P*>0.05) (**Figure 2C**).

### The ratio of Tim molecules and T-cell subset-specific transcription factor expression in the T1D patient subgroups

**Figure 3:**
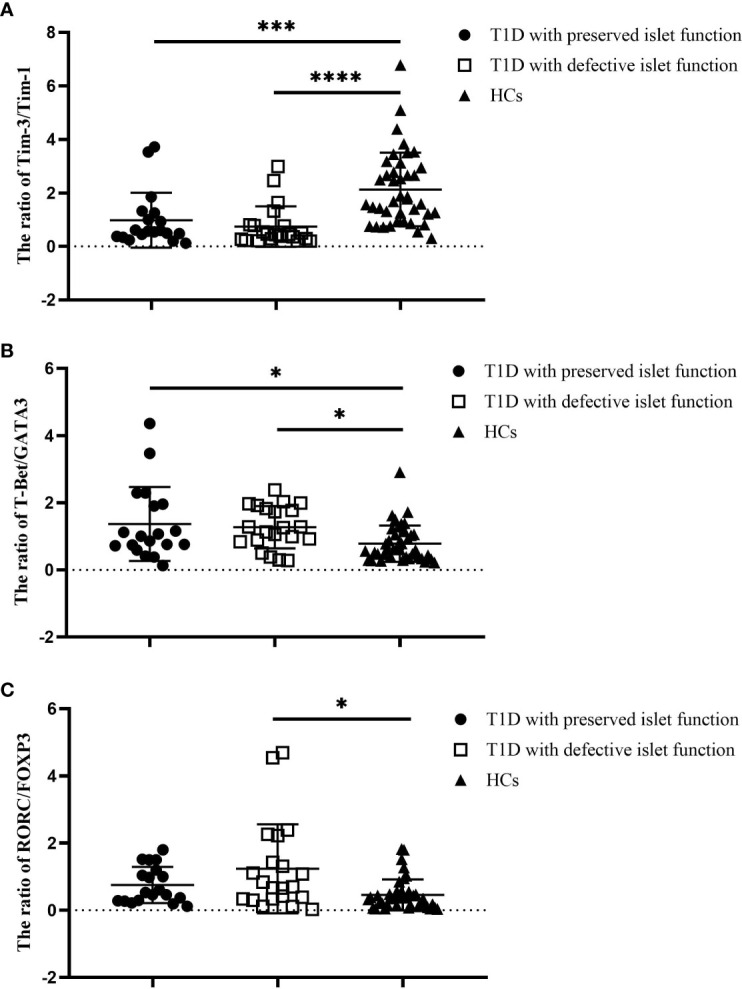
The ratios of Tim molecules and T-cell subset-specific transcription factor expression in the T1D subgroups (n=40) and HCs (n=40). **(A)** The ratio of Tim-3 to Tim-1 in different T1D subgroups and the HC group. **(B)** The ratio of T-Bet to GATA3 in different T1D patient subgroups and the HC group. **(C)** The ratio of RORC to POXP3 in different T1D subgroups and the HC group. Error bars represent the standard deviation of the mean. *P<0.05; ***P<0.001; ****P<0.0001.

To investigate the possible factors that influence the immune imbalance in T1D, We divided the T1D patients studied above into two subgroups based on their median FCP values. Nineteen T1D patients (10 males and 9 females) were defined as having preserved islet function, and their C-peptide values ranged from 102 to 365 pmol/L, with a median of 154 pmol/L. Twenty-one T1D patients (15 males and 6 females) were defined as having deficient islet function, and their C-peptide values ranged from 0 to 93 pmol/L, with a median of 55 pmol/L. We compared the expression ratios of Tim molecules and T-cell subset-specific transcription factors between these subgroups and HCs. The results show that compared with the HC group, the ratio of Tim-3/Tim-1 decreased in both the preserved islet function group (2.13 ± 1.38 vs. 0.98 ± 0.74, *P*=0.002) and the defective islet function group (2.13 ± 1.38 vs. 0.74 ± 0.76, *P*<0.0001) (**Figure 3A**). However, there was no significant difference between the two T1D subgroups (*P*>0.05). Second, the ratio of T-Bet/GATA3 expression was significantly increased in both T1D subgroups (0.78 ± 0.54 vs. 1.37 ± 1.10, *P*=0.014 and 0.78 ± 0.54 vs. 1.29 ± 0.63, *P=*0.039, respectively), and there was no significant difference between the two T1D subgroups (*P*>0.05) (**Figure 3B**). Finally, the RORC/FOXP3 ratio was reduced in the defective islet function group compared with the HC group (0.45 ± 0.46 vs. 1.24 ± 1.33, *P*=0.001). There was an increasing trend in the RORC/FOXP3 ratio in the preserved islet function group, but it did not reach statistical significance (*P*>0.05) (**Figure 3C**). The clinical characteristics of the T1D subgroups and HCs are shown in **Table S1**.

### Expression of Tim-1 and Tim-3 in T-cell subsets from T1D patients

**Figure 4:**
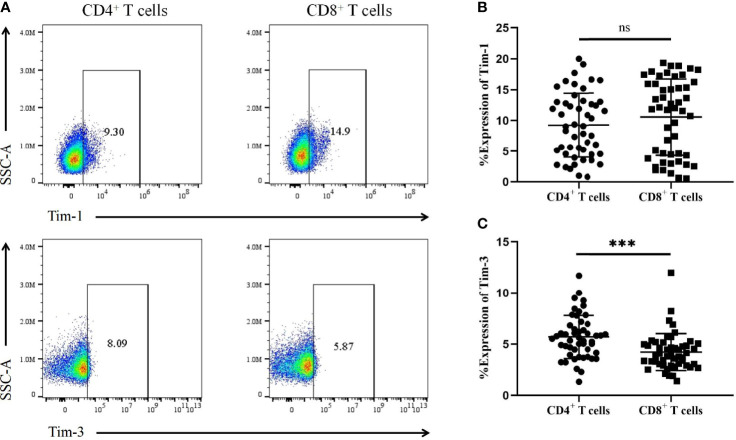
The expression of Tim-1 and Tim-3 in different T-cell subsets from T1D patients. **(A)** Representative plots showing the expression of Tim-1 and Tim-3 in different T-cell subsets. **(B)** The expression of Tim-1 on CD4^+^ and CD8^+^ T cells in T1D patients. **(C)** The expression of Tim-3 in CD4^+^ and CD8^+^ T cells in T1D patients. ns, no statistical difference; ***P<0.001.

We further analysed the expression of Tim-1 and Tim-3 in T-cell subsets from T1D patients (**Figure 4A**). The results showed no significant difference in Tim-1 levels on CD4^+^ T cells in T1D patients compared to CD8^+^ T cells (9.23 ± 5.19 vs. 10.52 ± 6.17, *P*=0.30) (**Figure 4B**). However, T1D patients had higher levels of Tim-3 on CD4^+^ T cells compared to those on CD8^+^ T cells (5.69 ± 2.10 vs. 4.22 ± 1.79, *P*=0.0003) (**Figure 4C**).

### Tim-1 and Tim-3 expression in T-cell subsets from T1D and T1D in the remission phase

**Figure 5:**
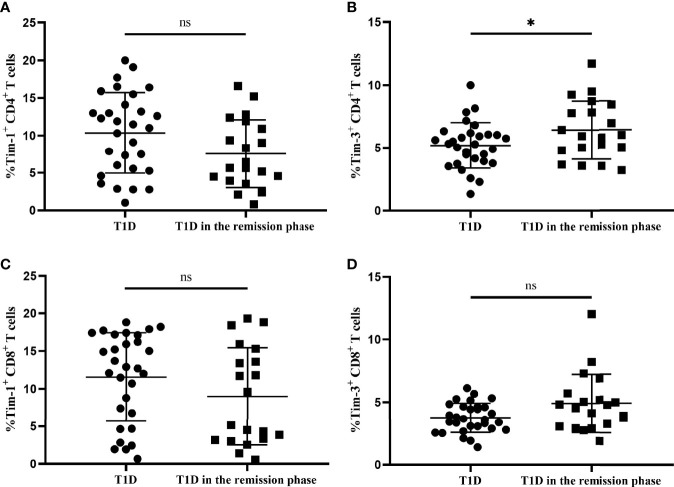
The Tim-1 and Tim-3 expression in T-cell subsets from T1D and T1D in the remission phase. **(A)** Alterations in the expression of Tim-1^+^ CD4^+^ T cells from T1D in the remission phase compared with T1D. **(B)** Alterations in the expression of Tim-3^+^ CD4^+^ T cells from T1D in the remission phase compared with T1D. **(C)** Alterations in the expression of Tim-1^+^ CD8^+^ T cells from T1D in the remission phase compared with T1D. **(D)** Alterations in the expression of Tim-3^+^ CD8^+^ T cells from T1D in the remission phase compared with T1D. ns, no statistical difference; *P<0.05.

We next compared the altered Tim-1 and Tim-3 expression in T1D and T1D in the remission phase. According to the preserved β-cell function, stimulated C-peptide above 300 pmol/L was defined as T1D in the remission phase, which is characterized by satisfactory glycaemic control and temporary recovery of islet β cells (20, 21). Our results showed that there was no significant difference in the expression of Tim-1 on CD4^+^ T cells between the two groups of T1D patients (7.56 ± 4.54 vs. 10.34 ± 5.37, *P*=0.063) (**Figure 5A**), whereas Tim-3 expression on CD4^+^ T cells from T1D in the remission phase was higher compared with T1D patients (6.43 ± 2.31 vs. 5.20 ± 1.82, *P*=0.043) (**Figure 5B**). In addition, there was no statistical difference in Tim-1 or Tim-3 expression in CD8^+^ T cells between both T1D patient groups (*P*>0.05 for both) (**Figures 5C, D**). The clinical characteristics of patients with T1D in both groups are summarized in **Table S2**.

### The Tim-3 to Tim-1 ratio from T1D and T1D in the remission phase

**Figure 6:**
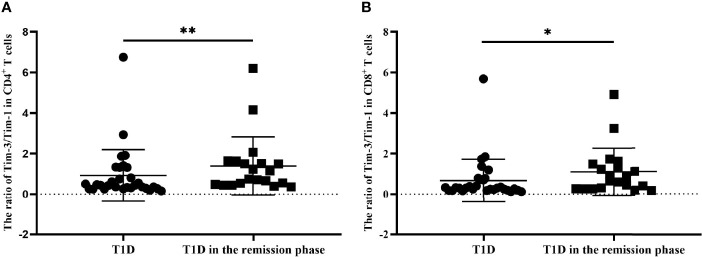
The ratio of Tim-3 to Tim-1 in different T-cell subsets form T1D and T1D in the remission phase. **(A)** The ratio of Tim-3 to Tim-1 in CD4^+^ T cells from T1D and T1D in the remission phase. **(B)** The ratio of Tim-3 to Tim-1 in CD8^+^ T cells from T1D and T1D in the remission phase. *P<0.05.;**P<0.01.

We finally explored the balance of Tim molecules on the different T-cell subsets from T1D and T1D in the remission phase. As is shown in **Figure 6**, the Tim-3 to Tim-1 ratio on CD4^+^ T cells from T1D in the remission phase was significantly increased compared to that in T1D (1.39 ± 1.43 vs. 0.92 ± 1.28, *P*=0.0087) (**Figure 6A**), while the ratio of Tim-3 to Tim-1 on CD8^+^ T cells was also increased in T1D during remission (1.10 ± 1.17 vs. 0.67 ± 1.06, *P*=0.0292) (**Figure 6B**).

## Discussion

Tim family molecules are involved in the regulation of the immune response, and they mainly regulate the proliferation of T cells (22). Currently, the pattern of Tim molecule expression in T1D patients is unknown. In our study, Tim and T-cell subset-specific transcription factor mRNA expression levels were altered in T1D and T2D patients compared with HCs. Imbalanced ratios of Tim-3/Tim-1, Th1/Th2 and Th17/Treg expression were identified in T1D patients. The imbalance in the ratio was more pronounced in T1D patients with defective islet function. Moreover, Tim-3 was mainly expressed on CD4^+^ T cells, and the expression of Tim-3^+^ CD4^+^ T cells in T1D in the remission phase was higher compared to that in T1D. In addition, there were differences in the ratio of Tim-3 to Tim-1 between T1D and T1D in the remission phase, suggesting that Tim molecules plays a role in the occurrence of T1D.

Wang et al. reported that Tim-1 expression was increased in SLE patients, especially in patients with active disease, indicating that Tim-1 expression may contribute to the degree of disease activity (23). Our published research found a decrease in Tim-1 expression in Breg cells in T1D patients (24). Another study showed that the frequency of Tim-1^+^ Tregs in both T1D patients and nonobese diabetic (NOD) mice was significantly decreased (25). Interestingly, Tim-1 mRNA expression was increased in the PBMCs from both T1D and T2D patients in this study. These discrepancies might result from the different patterns of Tim-1 expression in distinct cell types; PBMCs are heterogeneous, with the highest proportion of T cells, and Tim-1 plays an important role in T-cell activation (26). Unlike Tim-1, Tim-3 suppresses the Th1 immune response (27). We also examined Tim-3 expression in different T-cell subsets and found that Tim-3 was mainly expressed in CD4^+^ T cells. Interestingly, the frequency of Tim-3^+^ CD4^+^ T-cell expression was higher in T1D in the remission phase than in T1D. In general, CD8^+^ T cells primarily infiltrate islets in T1D, but their maturation and proliferation may require the promotion of CD4^+^ T cells (28). The reduced expression of Tim-3 in T1D patients indicates inadequate immune regulation, which may contribute to the pathogenesis of T1D. With respect to Tim-4, Zhao et al. found that the expression of Tim-4 in T2D patients was higher than that in healthy control, which is consistent with our findings (29). Although Tim-4 expression exhibited an increased trend in T1D patients, it is not statistically different compared with HCs.

The Th1/Th2 and Th17/Treg imbalance has been reported to be involved in the pathogenesis and progression of autoimmune diseases, including RA, MS and SLE (30–32). In this study, we evaluated the ratios of T-cell-specific transcription factor mRNA expression in all participants. As expected, we observed a significantly increased ratio of T-bet to GATA3 expression in T1D patients compared to HCs. In addition, the ratio of RORC to FOXP3 expression in T1D patients was also significantly higher than that in HCs. Our study suggested that T1D patients, especially those with defective islet function, exhibit an obvious imbalance in the RORC to FOXP3 ratio. However, the ratios of T-bet/GATA3 and RORC/FOXP3 in T2D patients were not significantly different compared with those of HCs, This discrepancy may be explained by the fact that the pathogeneses of these two types of diabetes are not quite consistent: T1D pathogenesis is mainly caused by immune imbalance, whereas T2D is a disease mediated by metabolic disorders (33).Notably, in a published study, Tim-3 on CD4^+^ T cells was negatively associated with Th1/Th2 imbalance: blockade of Tim-3 enhanced the production of Th1 responses such as IFN-γ and TNF-α, while it decreased Th2 responses (34). This finding suggests that Tim-3 expression in T1D patients may affect the Th1/Th2 balance and participate in the progression of T1D.

A relationship between Tim-3 and Tim-1 has been reported in autoimmune diseases (35, 36). Tim-1 is mainly expressed in Th2 cells and regulates the activation of T cells. In contrast, Tim-3 mainly inhibits Th1 cells thus acting as an anti-inflammatory molecule. The ratio of Tim-3 to Tim-1 expression was also investigated in the present study. We observed that the Tim-3 to Tim-1 ratio in PBMCs from T1D patients was significantly decreased, and this decrease was especially notable in the defective islet function group. In addition, the ratio of Tim-3 to Tim-1 in different T-cell subsets was increased in T1D in the remission phase compared with T1D, indicating an imbalance between pro- and anti-inflammatory properties in T1D patients. Hence, improving the balance between Tim-1 and Tim-3 might be a potential therapeutic approach for T1D.

There are several limitations of our study. First, some of the diabetes patients included had a long disease duration. Therefore, these results may be affected by factors specific to the included population and the medications used for treatment. Moreover, this study was a cross-sectional study without medical follow-up. Collectively, our study suggests that the expression of Tim family molecules is altered and may be involved in the pathogenesis of T1D.

## Data availability statement

The raw data supporting the conclusions of this article will be made available by the authors, without undue reservation.

## Ethics statement

The studies involving human participants were reviewed and approved by Ethics Committee of Second Xiangya Hospital, Central South University. The patients/participants provided their written informed consent to participate in this study. Written informed consent was obtained from the individual(s) for the publication of any potentially identifiable images or data included in this article.

## Author contributions

YL searched the references, wrote the first draft of the paper and revised the text. ZC and YX critically revised the text and provided substantial scientific contributions. ZZ and HC proposed the project and revised the manuscript. All the authors approved the final version of the manuscript.

## Funding

This study was supported by the National Key Research and Development Project (2018YFE0114500), National Science Foundation of China (81820108007, 81970746 and 31571244), Hunan Provincial Natural Sciences Foundation (2022JJ30797) and the Natural Science Foundation of China (82000748).

## Conflict of interest

The authors declare that the research was conducted in the absence of any commercial or financial relationships that could be construed as a potential conflict of interest.

## Publisher’s note

All claims expressed in this article are solely those of the authors and do not necessarily represent those of their affiliated organizations, or those of the publisher, the editors and the reviewers. Any product that may be evaluated in this article, or claim that may be made by its manufacturer, is not guaranteed or endorsed by the publisher.

## Supplementary material

Supplementary Figure 1 Representative plots and gating strategy for T-cell subsets from the flow cytometry analysis. **(A)** T-cell subsets were sequentially gated on lymphocytes, single cells, live cells and CD3^+^ T cells, and the expression of CD4 and CD8 markers on CD3^+^ T cells was further analysed. FSC-A, forward scatter area; FSC-H, forward scatter height; SSC-A, side scatter area.

Supplementary Table 1 Clinical characteristics of the T1D subgroups and the HC group. The data are expressed as the mean ± standard deviation or as the median of the 25th-75th percentile in parentheses. NA: not applicable. ^##^ *P*<0.01, compared with T1D with defective islet function. ^####^ *P*<0.0001, compared with T1D with defective islet function. ^**^ *P*<0.01, compared with HCs. ^***^ *P*<0.001, compared with HCs. ^****^ *P*<0.0001, compared with HCs.

Supplementary Table 2 Clinical characteristics of T1D and T1D in the remission phase. The data are expressed as the mean ± standard deviation or as the median of the 25th-75^th^ percentile in parentheses. ^**^ *P*<0.01, compared with T1D in the remission phase. ^***^ *P*<0.001, compared with T1D in the remission phase.
