# Supplementary figures and images for: Altered expression of Tim family molecules and an imbalanced ratio of Tim-3 to Tim-1 expression in patients with type 1 diabetes
Source: Front Endocrinol (Lausanne). 2022 Jul 28;13:937109. doi: 10.3389/fendo.2022.937109 (PMC9366857; doi:10.3389/fendo.2022.937109)

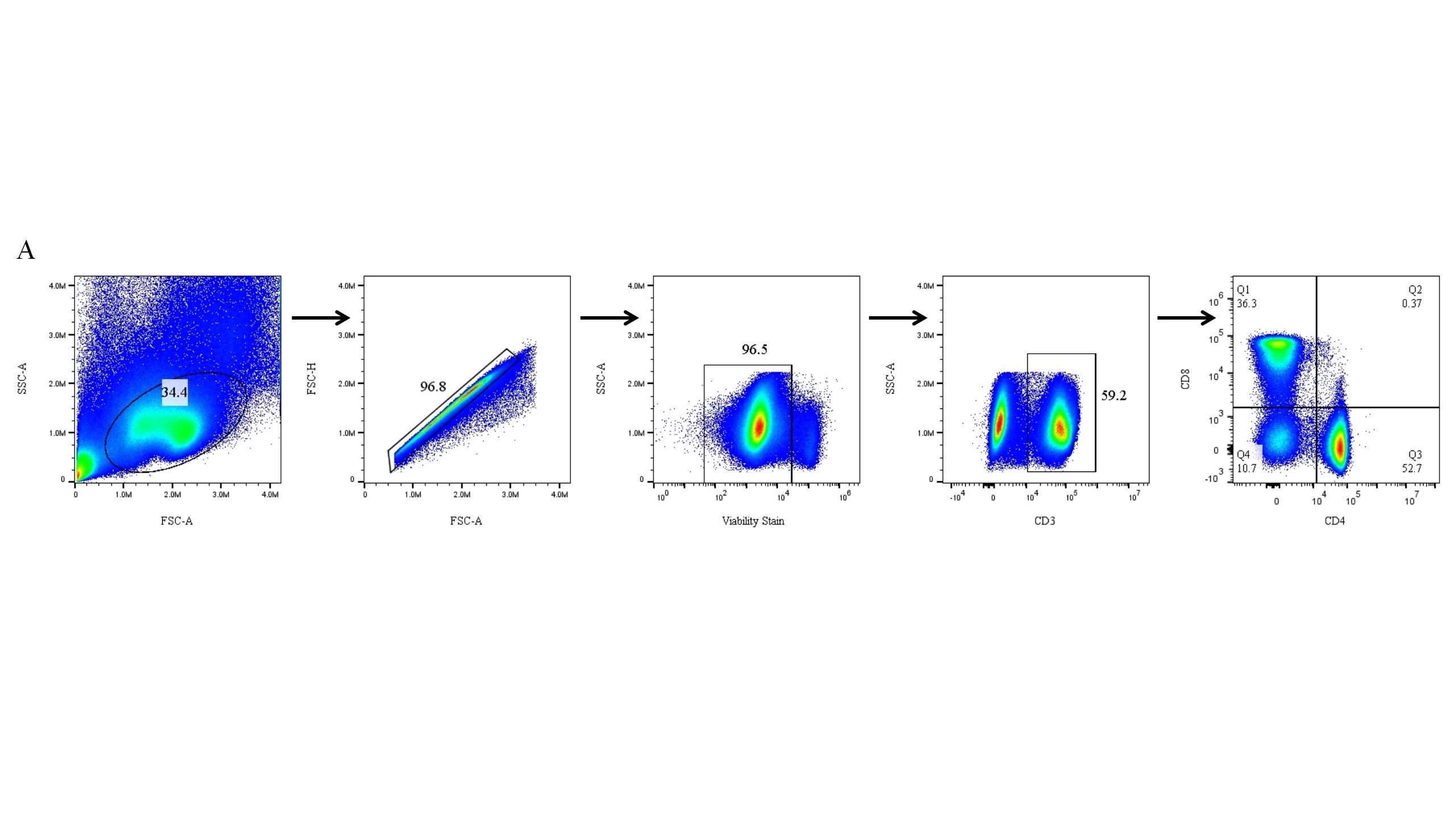

Supplement: Supplementary Figure 1 — Representative plots and gating strategy for T-cell subsets from the flow cytometry analysis. (A) T-cell subsets were sequentially gated on lymphocytes, single cells, live cells and CD3+ T cells, and the expression of CD4 and CD8 markers on CD3+ T cells was further analysed. FSC-A, forward scatter area; FSC-H, forward scatter height; SSC-A, side scatter area. [file Image_1.jpeg]
