# Supplementary material for: Altered expression of Tim family molecules and an imbalanced ratio of Tim-3 to Tim-1 expression in patients with type 1 diabetes
Source: Front Endocrinol (Lausanne). 2022 Jul 28;13:937109. doi: 10.3389/fendo.2022.937109 (PMC9366857; doi:10.3389/fendo.2022.937109)
Supplement: Supplementary Table 1 — Clinical characteristics of the T1D subgroups and the HC group. The data are expressed as the mean ± standard deviation or as the median of the 25th-75th percentile in parentheses. NA: not applicable. ## P<0.01, compared with T1D with defective islet function. #### P<0.0001, compared with T1D with defective islet function. ** P<0.01, compared with HCs. *** P<0.001, compared with HCs. **** P<0.0001, compared with HCs. [file Table_1.docx]

Table S1.

Clinical characteristics of the T1D subgroups and the HC group.

| **Variable** | **T1D with preserved islet function (n=19)** | **T1D with defective islet function (n=21)** | **HCs**  **(n=40)** |
| --- | --- | --- | --- |
| Sex (male/female) | 10/9 | 15/6 | 26/14 |
| Age (years) | 24.89±7.852 | 29.14±10.07 | 28.25±7.62 |
| BMI (kg/m^2^) | 20.70±1.64^**^ | 22.23±3.48 | 23.72±3.04 |
| Duration (months) | 19.58±13.07^##^ | 50.81±47.13 | NA |
| FBG (mmol/L) | 6.92 (5.59-8.56)^****^ | 8.43 (6.82-13.97)^****^ | 4.71 (4.34-4.97) |
| FCP (mmol/L) | 154.2 (135.5-190.6)^***##^ | 54.6 (17.55-68.1)^****^ | 357.1 (258.6-381.6) |
| PCP (mmol/L) | 334.2 (193.6-685.4) ^****^ | 63 (16.5-152.2) | NA |
| HbA1c (%) | 7.48±2.33^****^ | 8.42±2.12^****^ | 5.27±0.26 |
| TG (mmol/L) | 0.82 (0.63-0.93) | 0.77 (0.68-1.20) | 1.00 (0.70-1.83) |
| TC (mmol/L) | 4.86±1.15 | 4.70±1.21 | 4.70±1.04 |
| LDL-C (mmol/L) | 2.83±0.95 | 2.61±0.98 | 3.00±0.93 |
| HDL-C (mmol/L) | 1.66±0.40^**^ | 1.52±0.43 | 1.29±0.28 |
| GADA | 12/19 (63.2%) | 13/21 (61.9%) | NA |
| IA-2A | 14/19 (73.7%) | 7/21 (33.3%) | NA |
| ZnT8A | 6/19 (31.6%) | 7/21 (33.3%) | NA |

The data are expressed as the mean ± standard deviation or as the median of the 25th-75th percentile in parentheses. NA: not applicable.

^##^*P*<0.01, compared with T1D with defective islet function. ^####^*P*<0.0001, compared with T1D with defective islet function.

^**^*P*<0.01, compared with HCs. ^***^*P*<0.001, compared with HCs. ^****^*P*<0.0001, compared with HCs.
