# Supplementary material for: Altered expression of Tim family molecules and an imbalanced ratio of Tim-3 to Tim-1 expression in patients with type 1 diabetes
Source: Front Endocrinol (Lausanne). 2022 Jul 28;13:937109. doi: 10.3389/fendo.2022.937109 (PMC9366857; doi:10.3389/fendo.2022.937109)
Supplement: Supplementary Table 2 — Clinical characteristics of T1D and T1D in the remission phase. The data are expressed as the mean ± standard deviation or as the median of the 25th-75th percentile in parentheses. ** P<0.01, compared with T1D in the remission phase. *** P<0.001, compared with T1D in the remission phase. [file Table_2.docx]

Table S2.

Clinical characteristics of T1D and T1D in the remission phase.

| **Variable** | **T1D**  **(n=30)** | **T1D in the remission**  **phase (n=20)** |
| --- | --- | --- |
| Sex (male/female) | 21/9 | 13/7 |
| Age (years) | 24.93±15.29 | 23.50±11.30 |
| BMI (kg/m^2^) | 19.80±3.70 | 20.50±2.63 |
| Duration (months) | 27.90±22.21^**^ | 10.75±9.01 |
| FBG (mmol/L) | 8.65±4.67 | 6.97±1.90 |
| FCP (mmol/L) | 75.80 (16.50-154.1)^***^ | 203 (145.3-258.4) |
| PCP (mmol/L) | 122.7 (24.40-457.8)^***^ | 442.9 (325.9-831.3) |
| HbA1c (%) | 7.93±2.25 | 7.59±1.71 |
| TG (mmol/L) | 4.59 (3.73-5.23) | 4.02 (3.56-4.82) |
| TC (mmol/L) | 0.89±0.67 | 0.96±0.87 |
| LDL-C (mmol/L) | 2.60±0.75 | 2.40±0.86 |
| HDL-C (mmol/L) | 1.61±0.39 | 1.42±0.37 |
| CD4^+^T cells (%) | 43.40±10.90 | 45.73±12.23 |
| CD8^+^ T cells (%) | 34.19±9.69 | 34.38±9.78 |
| GADA | 19/30 (63.3%) | 16/20 (80%) |
| IA-2A | 18/30 (60%) | 16/20 (80%) |
| ZnT8A | 12/30 (40%) | 9/20 (45%) |

The data are expressed as the mean ± standard deviation or as the median of the 25th-75th

percentile in parentheses.

^**^*P*<0.01, compared with T1D in the remission phase.

^***^*P*<0.001, compared with T1D in the remission phase.
